# Trends of Antibiotic Use and Expenditure After an Intensified Antimicrobial Stewardship Policy at a 2,200-Bed Teaching Hospital in China

**DOI:** 10.3389/fpubh.2021.729778

**Published:** 2021-09-21

**Authors:** Xiaodan Qian, Yuyan Pan, Dan Su, Jinhong Gong, Shan Xu, Ying Lin, Xin Li

**Affiliations:** ^1^Department of Pharmacy, Changzhou No. 2 People's Hospital, The Affiliated Hospital of Nanjing Medical University, Changzhou, China; ^2^Department of Clinical Pharmacy, School of Pharmacy, Nanjing Medical University, Nanjing, China; ^3^Department of Health Policy, School of Health Policy and Management, Nanjing Medical University, Nanjing, China; ^4^Center for Global Health, School of Public Health, Nanjing Medical University, Nanjing, China

**Keywords:** antimicrobial stewardship, intensified intervention, antibiotic consumption, interrupted time series analysis, China

## Abstract

**Objective:** This study aimed to evaluate the effects of intensified Chinese special rectification activity on clinical antibiotic use (CSRA) policy on a tertiary-care teaching hospital.

**Methods:** A 48-month longitudinal dataset involving inpatients, outpatients, and emergency patients were collected. Study period included pre-intervention stage (adopting soft measures like systemic training) and post-intervention stage (applying antibiotic control system to intensify CSRA policy). Antibiotic use was evaluated by antibiotic use rate (AUR) or antibiotic use density (AUD). Economic indicator was evaluated by antibiotic cost in prescription or antibiotic expenditure in hospitalization. Data was analyzed by interrupted time series (ITS) analysis.

**Results:** The medical quality indicators remained stable or improved during the study period. AUR of inpatients (AURI) declined 0.553% per month (*P* = 0.025) before the intervention and declined 0.354% per month (*P* = 0.471) after the intensified CSRA policy was implemented. AUD, expressed as defined daily doses per 100 patients per day (DDDs/100PD), decreased by 1.102 DDDs/100PD per month (*P* = 0.021) before and decreased by 0.597 DDDs/100PD per month (*P* = 0.323) thereafter. The ratio of antibiotic expenditure to medication expenditure (AE/ME) decreased by 0.510% per month (*P* = 0.000) before and fell by 0.096% (*P* = 0.000) per month thereafter. AE per patient decreased by 25.309 yuan per month (*P* = 0.002) before and decreased by 7.987 yuan per month (*P* = 0.053) thereafter. AUR of outpatient (AURO) decreased by 0.065% per month before (*P* = 0.550) and decreased by 0.066% per month (*P* = 0.994) thereafter. The ratio of antibiotic cost to prescription cost in outpatient (ACO/PCO) decreased by 0.182% per month (*P* = 0.506) before and decreased by 0.216% per month (*P* = 0.906) thereafter. AUR of emergency patient (AURE) decreased by 0.400% per month (*P* = 0.044) before and decreased by 0.092% per month (*P* = 0.164) thereafter. The ratio of antibiotic cost to prescription cost in emergency patient (ACE/PCE) decreased by 0.616% per month (*P* < 0.001) before and decreased by 0.151% per month (*P* < 0.001) thereafter.

**Conclusions:** Implementation of CSRA policy was associated with declining antibiotic use and antibiotic expenditure in inpatients, outpatients, and emergency patients. However, it is also important to note that the declining trend of antibiotic consumption slowed due to the limited capacity for decline in the later stages of CSRA intervention.

## Background

Antibiotics have been widely used for more than 70 years. During this time, inappropriate or excessive use of antibiotics has accelerated antibiotic resistance. Substantial reports have demonstrated the causal relationships between antibiotic consumption and selective pressure in resistant organisms (1–4). Hence, strengthening antimicrobial stewardship (AMS) has become a global healthcare concern. In 2007, the Infectious Diseases Society of America published the Guidelines for Developing an Institutional Program to Enhance Antimicrobial Stewardship. These guidelines were the first to conceptualize AMS as a set of interventions targeted toward addressing the rapid development of antibiotic resistance in hospital settings. Thereafter, many countries joined the AMS program and achieved positive outcomes (5–8).

The overuse of antibiotics is also a serious problem in China (9). The percentage of antibiotic prescriptions for inpatients reached 70% (10), although the target recommended by the World Health Organization is below 30% (11). A high rate of antibiotic prescription also occurs in outpatient cases (12, 13). The antibiotic use density (AUD), expressed as defined daily doses per 100 patients per day (DDDs/100PD), increased to 80.1 DDD/100 patient days before 2011 in China, which is much higher than the average AUD worldwide of >40 DDDs/100PD (14). Given these facts, focused strategies are necessary to reduce antibiotic use in China. Since 2009, the government has reissued a series of policies and regulations to address this issue. The most stringent intervention was the Chinese Special Rectification Activity on Clinical Antibiotic Use (CSRA), which was initiated in 2011. Thirteen detail-oriented measures concerning management, procurement, and usage of antibiotics were proposed. CSRA guidelines were implemented nationwide for three consecutive years from 2011 to 2014.

Many Chinese studies have evaluated the impact of CSRA guidelines on antibiotic use in hospitals and affirmed the positive outcomes yielded by this policy (15–18). However, most prior studies were cross-sectional analyses or pre- post-comparisons at two time points that could not reveal the dynamics of particular variables over time. Such studies may not be able to fully examine the potential effects of CSRA. Moreover, most of these studies were published in Chinese journals, reducing the dissemination of their contents to a global audience. Only two studies about CSRA were retrieved from databases in a foreign language, and each had a different slant to it. For example, Zou et al. (2) reported a correlation between antibacterial usage and bacterial resistance after CSRA, while another study showed the impact of CSRA guidelines on reducing multidrug-resistant organism isolates in critically ill patients (19). Overall, more empirical research is needed to deepen our understanding of the impacts of CSRA guidelines and effectively communicate these findings with the global community.

Changzhou No. 2 People's Hospital, a 2,200-bed tertiary general hospital affiliated with a medical university in Jiangsu, began to implement CSRA guidelines in 2011. First, the implementation of the policy consisted of soft measures such as founding an AMS team, implementing systemic training, and strengthening the education of healthcare providers. By April 2012, an antibiotic control system (ACS) was constructed in this hospital which integrated specific targets of the CSRA policy into the original Hospital Information System (HIS). Rigid measures have been deployed over the entire hospital to intensify the AMS since April 2012.

Here, we conducted an interventional study in this hospital from 2011 to 2014, with a focus on how antibiotic use and economic indicators differed before and after the intensified intervention. Combined with interrupted time series (ITS) analysis, the strongest and quasi-experimental approach for evaluating longitudinal effects of intervention (20), we aimed to obtain a robust estimation of the effects of an intervention to offer constructive policy suggestions to improve managerial decisions regarding antibiotics.

## Methods

### Setting and Study Design

The study was conducted in Changzhou No. 2 People's Hospital, a 2,200-bed general tertiary-care teaching hospital in Jiangsu, China. The annual number of outpatient and emergency visits to the hospital is more than 2.39 million, and the annual number of discharged patients is 102.4 thousand. As a result, this hospital has a significant impact on Changzhou. Changzhou lies to the south of Jiangsu province. It is located in the affluent Yangtze Delta region of China, halfway between Shanghai and Nanjing. The city covers an area of 4,385 square kilometers and has a permanent resident population of 5.27 million people. In 2020, the GDP of this city reached 780.53 billion yuan.

As described in the introduction, Changzhou No. 2 People's Hospital began to implement the CSRA policy in 2011. First, the implementation of the policy only consisted of soft measures. By April 2012, the ACS was constructed, and rigid measures have been deployed over the entire hospital to intensify CSRA policy. To evaluate the impacts of intensified CSRA policy on this hospital and even on local patients, we conducted a 48-month, retrospective time series analysis study in this hospital. Data related to outpatients, emergency patients, and inpatients were retrieved from the HIS between January 2011 and December 2014 in hospital-wide population-level monthly. The pre-intervention period was defined as from January 2011 to March 2012 (15 months in total), and the post-intervention period was defined as from April 2012 to December 2014 (33 months in total).

### Intensified CSRA Policy

First, we briefly introduce the Chinese special rectification activity on clinical antibiotic use (CSRA) initiated by the Chinese government in 2011. This policy strengthens the clinical antibiotic management in thirteen detailed ways. The five most important ones are as follows: (1) antibiotics are categorized into three classifications, namely, unrestricted, restricted, and special level. For each level of antibiotics, they can only be prescribed by clinicians who have been authorized according to their technical titles; (2) limit the number of antibiotics used in hospitals. In a general tertiary hospital, the total number of unique antibiotic cannot exceed 50; (3) set a specific target for antibiotic use. Antibiotic use rate (AUR) of inpatients, emergency patients, and outpatient cannot exceed 60, 40, 20%, respectively. AUD should be controlled under 40 DDDs/100PD in a general tertiary hospital; (4) microbial specimen detection is required before antibiotic use and detection rate of inpatients must exceed 30%; (5) vetting exercises should be carried out periodically by infectious disease experts or clinical pharmacists. In response to the policy of the government, Changzhou No. 2 People's Hospital began to implement the CSRA policy in 2011. At first, soft measures such as founding an AMS team (including infectious disease professionals, clinical microbiologists, pharmacists, and nurses), systemic training, and education were only adopted. Thus, we defined the period from January 2011 to March 2012 as pre-intensified policy stage.

Here, we emphatically introduced the intensified CSRA policy. In April 2012, rigid measures were deployed over the entire hospital to intensify AMS. We developed an ACS and integrated it into HIS. In ACS, the classification of antibiotics and prescription authorization are computerized. Data related to AUR, AUD, and microbial specimen detection rates of each ward can be easily retrieved from the ACS. Clinicians in each ward would receive bonuses if they met the quota, or penalties if the quota was not met. In addition, for each inappropriate prescription, there was a penalty of 50 yuan, and for each inappropriate medical record, the penalty increased to 500 yuan. In short, ACS, along with awards and penalties, offered better opportunities and incentives to optimize antibiotic use. Thus, we called it intensified CSRA policy. We defined the period from April 2012 to December 2014 as post-intensified policy stage.

### Study Outcomes

In this study, four items, namely, antibiotic use rate of inpatients (AURI), AUD, the ratio of antibiotic expenditure to medication expenditure (AE/PE), and antibiotic expenditure per patient (AE per patient) were selected as indicators of inpatient antibiotic use. AUD is defined as the number of defined daily doses/100 patient-days (DDDs/100PD) according to the anatomical therapeutic chemical (ATC)/DDD classification stated by WHO. DDD is the average daily dose in grams of a specific agent given to an average adult patient.

We selected three indicators to evaluate antibiotic use of outpatients, namely, antibiotic use rate of outpatients (AURO), antibiotic cost of outpatient/total prescription cost of outpatient (ACO/PCO), and antibiotic cost per patient in outpatient (ACO per patient).

Similarly, we selected three indicators to evaluate antibiotic use of emergency patient which include antibiotic use rate of emergency patients (AURE), antibiotic cost of emergency patient/total prescription cost of emergency patient (ACE/PCE), and antibiotic cost per patient in emergency patient (ACE per patient).

The values of outcome variables were calculated at the hospital level every month, and the measurement is explained in detail in Table 1.

**Table 1:** Study outcome variables.

**Classification**	**Indicators**	**Measurement**	**Units of measurement**
Inpatient	antibiotic use rate of inpatients (AURI)	the number of discharged patients who used antibiotics/the total number of discharged patients	Percentage
	antibiotic use density (AUD)	the usage of all kind of antibiotics calculated by defined daily dose (DDD)^a^ ×100/(number of patients × length of stay per patient)	DDDs/100PD
	AE/ME	antibiotic expenditure (AE)/total medication expenditure(ME)	Percentage
	AE per patient	total antibiotic expenditure/the number of discharged patients	RMB^b^
Outpatient patient	antibiotic use rate of outpatients (AURO)	the number of outpatients who used antibiotics/the total number of outpatients	Percentage
	ACO/PCO	antibiotic cost of outpatients (ACO)/prescription cost of outpatients (PCO)	Percentage
	ACO per patient	antibiotic cost of outpatients (ACO)/the total number of outpatients	RMB
Emergency patient	antibiotic use rate of emergency patients (AURE)	the number of emergency patients who used antibiotics/the total number of emergency patients	Percentage
	ACE/PCE	antibiotic cost of emergency patients (ACE)/prescription cost of emergency patients (PCE)	Percentage
	ACE per patient	antibiotic cost of emergency patients (ACE)/the total number of emergency patients	RMB

a *DDD is stated by the WHO*.

b *RMB is the abbreviation of renminbi, a Chinese currency*.

During the study period, medical quality indicators were also captured in hospital-wide population level every year. They were the number of hospitalizations, patient-days, recovery rate, improvement rate, invalid rate, mortality rate, and nosocomial infection rate.

### Statistical Analysis

We briefly describe ITS analysis, the strongest quasi-experimental approach, for evaluating the longitudinal effects of intervention when a control group is unavailable or unethical (20). This type of study design can reduce threats to internal validity and obtain richer information from the data.

**Figure 1:**
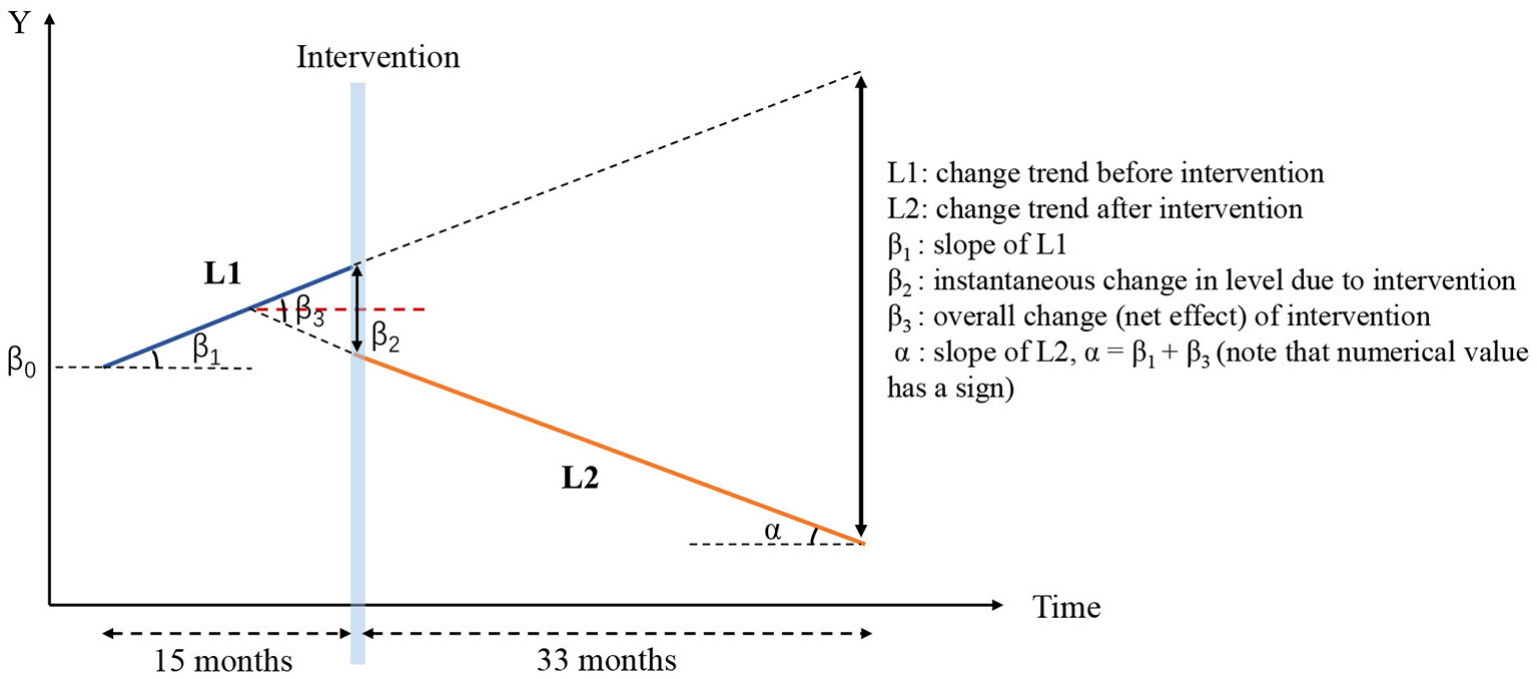
Graphical illustration of interrupted time series analysis model in this study.

In this model, the results of each variable are presented in three parts as described in our previous study (21). They are as follows: (1) slope for the pre-intervention period, (2) instantaneous change in level for the post-intervention period based on pre-intervention trend, and (3) change in slope from pre-intervention to post-intervention. The formula of the model is as follows:


$$\begin{array}{l} {\text{Y}}_{\text{t}}=\beta_{0}+\beta_{1}\text{T}+\beta_{2}\text{D}+\beta_{3}\text{P}+\varepsilon \end{array}$$

where *Y* is the main outcome indicator, such as the AURI; *T* is a continuous time variable, assigning 1–48 in this study; *D* is a dummy variable assigned a value of 0 before intervention and 1 after the intervention; *P* is a time variable counting the number of months after the intervention—for the time before the intervention, *P* = 0, and after the intervention, P is assigned a value from 1 to 34 in this study; ε is a random variable which cannot be explained in the linear model, which we designate the “error term”; β_0_ is the baseline level of the outcomes when *T* = 0; β_1_ is an estimate of changing trends during the pre-intervention period; β_2_ is the level of changes in the policy intervention; β_3_ is the trend change of the outcome caused by the policy intervention. Here, β_1_ + β_3_ represents the final trend of the outcome variable after policy intervention, which we call the net effect. A graphical illustration of the ITS model is shown in Figure 1.

Linear regression was carried out in order to assess the changes in trends of medical quality indicators during the study period. The linear trend by year is defined as the slope of the response over time, expressed by a coefficient (β).

The Durbin–Watson test is the most commonly used method for testing first-order autocorrelation of data (22). Generally, autocorrelation exists if the Durbin–Watson statistic approaches 0 or 4. Conversely, data are independent if the Durbin–Watson statistic is close to 2. The feasible generalized least square (FLGS) method can be used to adjust the first-order autocorrelation errors if needed (23–25). Statistical analysis was performed using STATA v.14 software (STATA Corporation, College Station, TX, USA). Continuous variables are summarized as means and standard deviations. Statistical significance was confirmed at a *p*-value of ≤ 0.05.

## Results

### Medical Quality Indicators

As shown in Table 2, medical quality indicators were captured regarding the number of hospitalizations, patient-days, recovery rate, improvement rate, invalid rate, mortality rate, and nosocomial infection rate. During and after the study period, the number of hospitalizations and improvement rate significantly increased annually (β = 9.838 and 2.112, *P* = 0.011 and 0.012, respectively). Simultaneously, the average length of stay and recovery rate both significantly decreased (β = −0.36 and −1.94, *P* = 0.013 and 0.023, respectively), and the incidence of mortality mildly decreased (β = −0.041, *P* = 0.012). There was no significant change in the invalid rate (β = −0.135, *P* = 0.226). Meanwhile, the nosocomial infection rate appeared to be declining, however, the results was not statistically significant (β = −0.107, *P* = 0.623). In summary, medical quality indicators remained stable or improved during and after the implementation of CSRA policy.

**Table 2:** Annual medical quality indicators in the Changzhou No. 2 People's Hospital, 2011–2015.

**Medical quality indicators**	**2011**	**2012**	**2013**	**2014**	**2015**	**Time series analysis model**		
						**β**	***P***	**Trend**
Number of hospitalizations (1,000 patients)	30.89	50.35	58.91	67.78	71.36	9.838	0.011	Increasing
Average length of stay (Days)	11.3	11.1	10.3	10.1	10.0	−0.36	0.013	Decreasing
Recovery rate (%)	34.62	30.00	28.10	28.36	25.74	−1.94	0.023	Decreasing
Improvement rate (%)	63.21	67.29	69.85	69.81	72.51	2.112	0.012	Increasing
Invalid rate (%)	1.92	2.44	1.87	1.67	1.63	−0.135	0.226	Stable
Mortality rate (%)	0.26	0.27	0.18	0.16	0.11	−0.041	0.012	Decreasing
Nosocomial infection rate (%)	1.62	2.99	2.10	1.88	1.64	−0.107	0.623	Stable

### Overall Changes in Antibiotic Use Due to Intensified CSRA Policy

#### Overall Changes in Antibiotic Use in Inpatients Before and After Intervention

Table 3 shows the overall change in the four indicators with respect to inpatient antibiotic use before and after the intervention. AURI decreased from 70.23% to 58.05% (*P* < 0.001) after the implementation of the intensified CSRA policy. AUD dropped from 87.02 to 58.26 DDDs/100PD (*P* < 0.001) after intervention. AE/ME was significantly decreased, from 24.04 to 17.49% (*P* < 0.001). Moreover, AE per patient decreased, resulting in a savings of 322.56 yuan per patient after intervention (*P* < 0.001). Overall, the intensified CSRA policy had a positive effect on reducing antibiotic use in inpatients.

**Table 3:** Overall changes of four indicators representing inpatient antibiotic use.

Indicators^*^	**Before intervention**	**After intervention**	***P-*Value**
AURI (%)	70.23	58.05	<0.001
AUD (DDDs/100PD)	87.02	58.26	<0.001
AE/ME (%)	24.04	17.49	<0.001
AE per patient (yuan)	1238.72	916.16	<0.001

* *The indicators in this table were calculated on the average of the monthly data*.

*AURI, antibiotic use rate of inpatients; AUD, antibiotic use density, expressed as defined daily doses per 100 patients per day (DDDs/100PD); AE, antibiotic expenditure; ME, medication expenditure*.

#### Overall Changes in Antibiotic Use in Outpatients Before and After Intervention

The overall changes in the three indicators with regard to antibiotic use in outpatient are displayed in Table 4. AURO decreased from 24.95 to 21.87% after intensified CSRA policy was implemented (P <0.001). ACO contributed to 42.68% of PCO before intervention, and decreased to 39.06% after intervention (P = 0.006). Unexpectedly, ACO per patient increased by 36.05 yuan when compared with the pre-intervention period, and this difference was statistically significant (P <0.001). In summary, the intensified CSRA policy reduced AURO, but increased antibiotic cost per patient when using a two-time points comparison.

**Table 4:** Overall changes of three indicators representing antibiotic use in outpatient.

Indicators^*^	**Before intervention**	**After intervention**	***P-*Value**
AURO (%)	24.95	21.87	<0.001
ACO/PCO (%)	42.68	39.06	0.006
ACO per patient (yuan)	158.71	194.76	<0.001

* *The indicators in this table were calculated on the average of the monthly data*.

*AURO, antibiotic use rate of outpatients; ACO, antibiotic cost of outpatients; PCO, prescription cost of outpatients*.

#### Overall Changes in Antibiotic Use in Emergency Patients Before and After Intervention

The overall changes in the three indicators with regard to antibiotic use in emergency patients are displayed in Table 5. There was a significant decline in AURE after intensified CSRA policy intervention, from 64.71 to 55.10% (P <0.001). ACE/PCE dropped from 67.80 to 58.44% (P <0.001). ACE per patient is as much of a surprise as ACO per patient, because it increased by 5.51 yuan when compared to the pre-intervention period (P = 0.006). In summary, the intensified CSRA policy reduced AURE, but increased antibiotic cost per patient when using a two-time points comparison.

**Table 5:** Overall changes of three indicators representing antibiotic use in emergency patient.

Indicators^*^	**Before intervention**	**After intervention**	***P-*Value**
AURE (%)	64.71	55.10	<0.001
ACE/PCE (%)	67.80	58.44	<0.001
ACE per patient (yuan)	117.23	122.74	0.006

* *The indicators in this table were calculated on the average of the monthly data*.

*AURE, antibiotic use rate of emergency patients; ACE, antibiotic cost of emergency patients; PCE, prescription cost of emergency patients*.

### ITS Analysis of Inpatient Antibiotic Use

**Figure 2:**
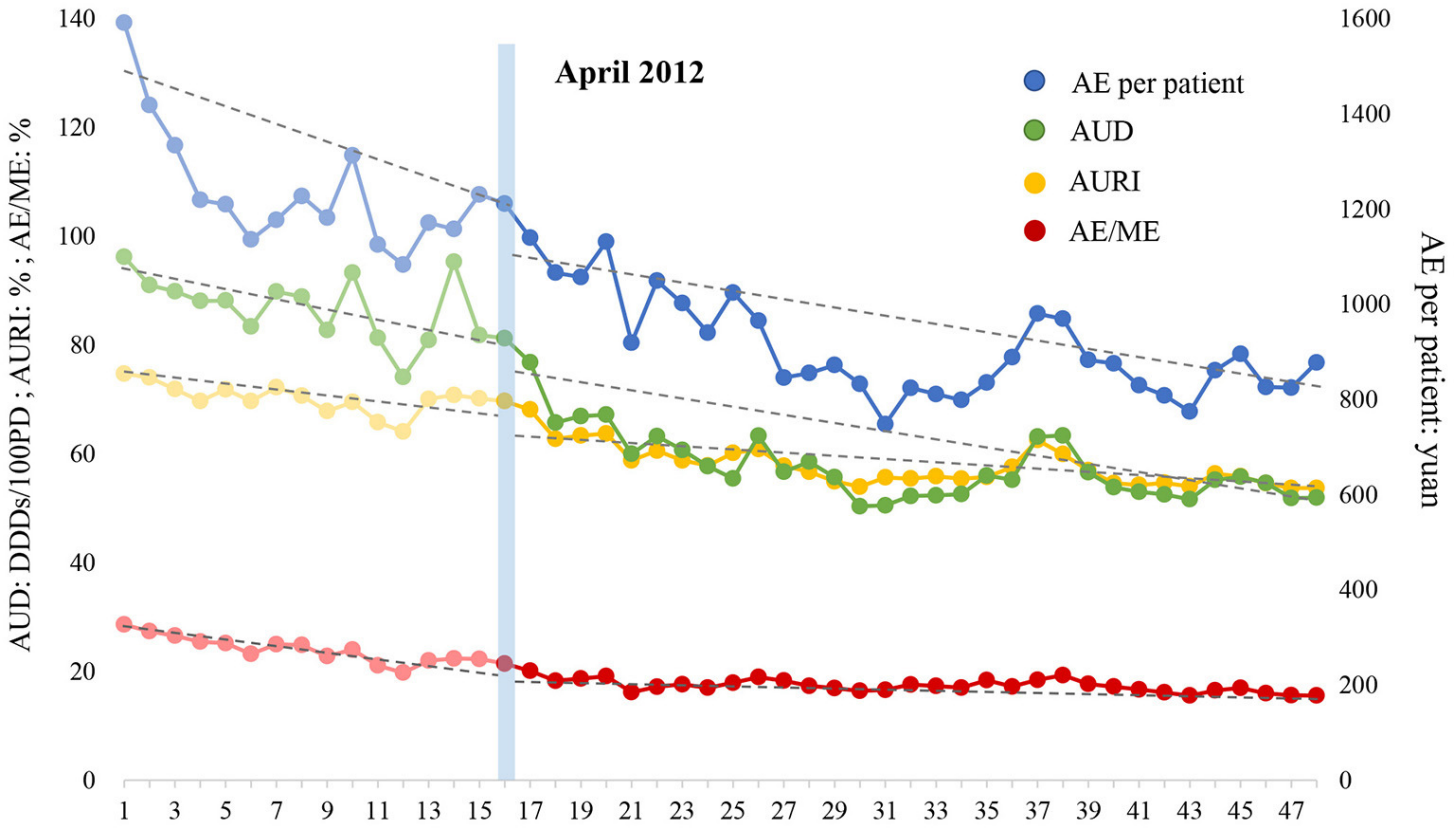
Effect of intensified CSRA policy on four indicators representing inpatient antibiotic use. AURI, antibiotic use rate of inpatients; AUD, antibiotic use intensity, expressed as defined daily doses per 100 patients per day (DDDs/100PD); AE, antibiotic expenditure; ME, medication expenditure.

The ITS analysis and the changing trend of four indicators with regard to inpatient antibiotic use are displayed in Table 6 and Figure 2, respectively. (1) AURI: AURI declined at a rate of 0.553% (P = 0.025) per month before the intensified policy was implemented. At the beginning of the intervention, the AURI dropped by 1.647% instantly (P = 0.426). However, the trend of AURI slowed down after the intensified policy was executed, decreasing at 0.354% (P = 0.471) per month. (2) AUD: AUD decreased by 1.102 DDDs/100PD (P = 0.021) per month before the intensified CSRA policy was implemented. At the beginning of the intervention, the AUD fell quite dramatically to a level of 9.419 DDDs/100PD (*P* = 0.037). However, after that, the downward trend gradually slowed down, decreasing at 0.597 DDDs/100PD (*P* = 0.323) per month. (3) AE/ME: AE/ME decreased before the intensified CSRA policy was implemented, and the rate of decrease was 0.510% per month (*P* = 0.000). The ratio dropped by 1.344% (*P* = 0.123) in the first month of intervention. Subsequently, AE/ME fell at a rate of 0.096% (*P* = 0.000) per month, which was significantly lower than that in the previous period. (4) AE per patient: AE per patient decreased at the rate of 25.309 yuan (*P* = 0.002) per month during the pre-intervention period. After the intensified CSRA policy was executed, no significant decline was noted immediately. In the post-intervention period, AE per patient decreased by 7.987 yuan (*P* = 0.053) per month, and the rate was significantly lower than before. In general, antibiotic use and expenditure in inpatients notably decreased during the pre-intervention period and the declining trend gradually tapered after the intensified CSRA policy was implemented.

**Table 6:** ITS analysis of inpatient antibiotic use.

**Indicators**	***β_1_*(SE)**	***P-*value**	***β_2_*(SE)**	***P-*value**	***β_3_*(SE)**	***P-*value**	***β_1_ + β3***	**Parameters of model fit**		
								**Dw**	**Root MSE**	***R* ^**2**^**
AURI (%)	−0.553 (0.237)	**0.025**	−1.647 (2.049)	0.426	0.199 (0.274)	0.471	−0.354	1.861	2.079	0.875
AUD (DDDs/100PD)	−1.102 (0.459)	**0.021**	−9.419 (4.390)	**0.037**	0.505 (0.505)	0.323	−0.597	2.085	4.944	0.799
AE/ME (%)	−0.510 (0.088)	**0.000**	−1.344 (0.855)	0.123	0.414 (0.096)	**0.000**	−0.096	1.888	0.996	0.881
AE per patient (yuan)	−25.309 (7.691)	**0.002**	−18.600 (69.727)	0.791	17.322 (8.707)	0.053	−7.987	1.976	73.177	0.771

*AURI, antibiotic use rate of inpatients; AUD, antibiotic use intensity, expressed as defined daily doses per 100 patients per day (DDDs/100PD); AE, antibiotic expenditure; ME, medication expenditure; Dw, Durbin-Watson. Bold values mean P <0.05 and they are statistically significant*.

### ITS Analysis of Antibiotic Use in Outpatient

**Figure 3:**
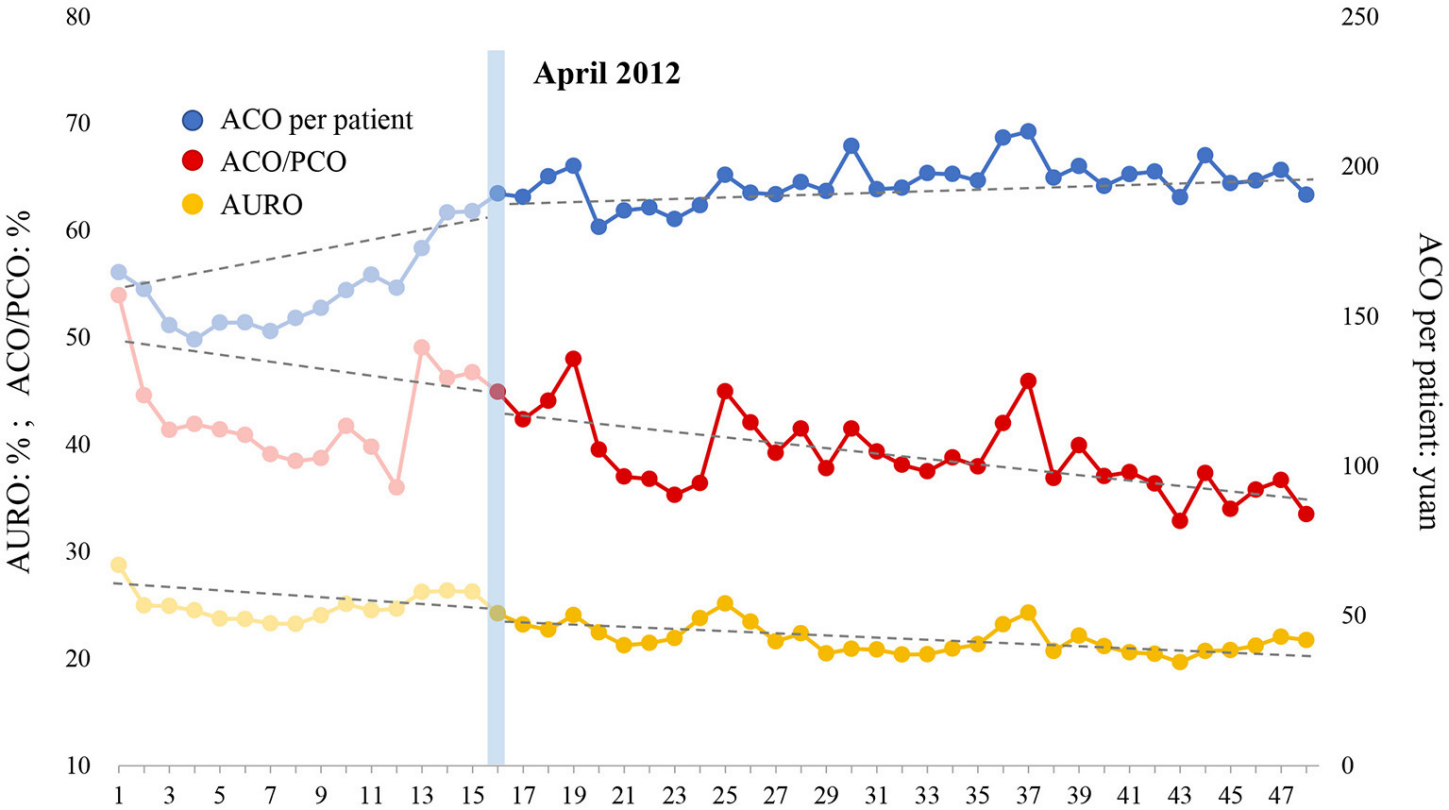
Effect of intensified CSRA policy on three indicators representing antibiotic use of outpatient patient. AURO, antibiotic use rate of outpatients; ACO, antibiotic cost of outpatients; PCO, prescription cost of outpatients.

Table 7 shows the results of three indicators with regard to outpatient antibiotic use estimated by the ITS model, and Figure 3 illustrates the overall trend of these three indicators. (1) AURO: AURO decreased at the rate of 0.065% (*P* = 0.550) per month before the intervention. An immediate drop of 1.731% (*P* = 0.097) was observed at the beginning month of intervention. After the intervention, the decreasing trend seemed to become slightly sharper at a rate of 0.066% (*P* = 0.994) per month, although this decrease was not statistically significant. (2) ACO/PCO: ACO/PCO declined at a rate of 0.182% (*P* = 0.506) per month before the intensified policy was implemented. The ratio instantly increased by 0.892% (*P* = 0.744) in the first month of intervention. After the intervention, the declining trend accelerated to a rate of 0.216% (*P* = 0.906) per month. (3) ACO per patient: Surprisingly, ACO per patient increased at a rate of 1.965 yuan per month (*P* = 0.003) before the intervention. At the beginning of the intervention, this indicator increased to a level of 14.623 yuan (P = 0.021) immediately. After that, the increasing trend of ACO per patient slowed down, increased by 0.320 yuan (P = 0.021) per month. In general, AURO and ACO/PCO gradually decreased before the intervention and the intensified CSRA policy accelerated the downward trend. Although ACO per patient significantly increased during the pre-intervention period, the intensified CSRA policy curbed the rapid increase of this indicator.

**Table 7:** ITS analysis of antibiotic use in outpatient patient.

**Indicators**	**β_1_(SE)**	**P-value**	**β_2_(SE)**	**P-value**	**β_3_(SE)**	**P-value**	**β_1_+ β_3_**	**Parameters of model fit**		
								**Dw**	**Root MSE**	**R^**2**^**
AURO (%)	−0.065 (0.108)	0.550	−1.731 (1.022)	0.097	−0.001 (0.120)	0.994	−0.066	1.805	1.122	0.761
ACO/PCO (%)	−0.182 (0.272)	0.506	0.892 (2.714)	0.744	−0.034 (0.291)	0.906	−0.216	1.834	3.489	0.468
ACO per patient (yuan)	1.965 (0.630)	**0.003**	14.623 (6.118)	**0.021**	−1.645 (0.687)	**0.021**	0.320	1.826	7.131	0.723

*AURO, antibiotic use rate of outpatients; ACO, antibiotic cost of outpatients; PCO, prescription cost of outpatients; Dw, Durbin-Watson. Bold values mean P <0.05 and they are statistically significant*.

### ITS Analysis of Antibiotic Use in Emergency Patient

**Figure 4:**
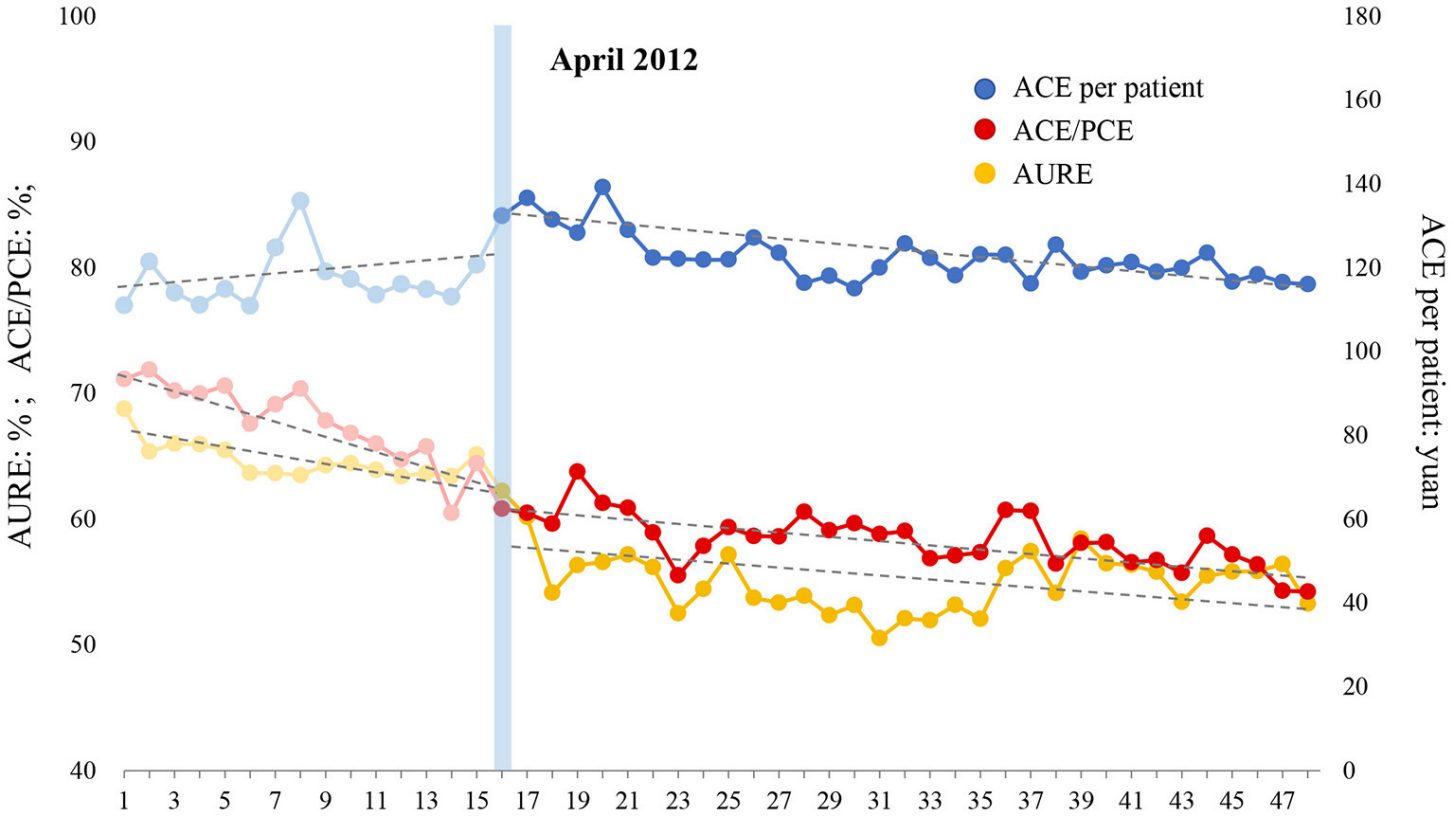
Effect of intensified CSRA policy on three indicators representing antibiotic use of emergency patient. AURE, antibiotic use rate of emergency patients; ACE, antibiotic cost of emergency patients; PCE, prescription cost of emergency patients.

The ITS analysis results and the changing trend of antibiotic use with regard to emergency patients are shown in Table 8 and Figure 4, respectively. (1) AURE: Before the intensified CRSA policy was implemented, AURE was reduced by 0.400% (*P* = 0.044) per month. At the beginning month of the intervention, there was a notable decrease in the level of 4.817% (*P* = 0.009). After the intervention, the declining trend gradually slowed down, decreasing by 0.092% (P = 0.164) per month. (2) ACE/PCE: ACE/PCE decreased before the intervention, and the rate of decrease was 0.616% (P <0.001) per month. At the beginning of the intervention, the ratio dropped greatly by 2.492% instantly (P = 0.020). However, the downward trend slowed down after the intervention, and decreased at a rate of 0.151% (P <0.001) per month. (3) ACE per patient: ACE per patient increased at a rate of 0.208 yuan (P = 0.591) per month before the intervention. At the beginning month of the intervention, this indicator increased to a level of 11.398 yuan (P = 0.005) immediately. After that, ACE per patient began to decrease at a rate of 0.422 yuan (P = 0.133) per month. In general, AURE and ACE/PCE notably decreased before the intervention, and the declining trend gradually tapered after the intensified CSRA policy was implemented. Although ACE per patient increased during the pre-intervention period, intensified CSRA policy stopped this increasing trend and showed a positive effect on reducing the antibiotic cost in the emergency patient.

**Table 8:** ITS analysis of antibiotic use in emergency patient.

**Indicators**	***β_1_*(SE)**	***P*-value**	***β_2_*(SE)**	***P-*value**	***β_3_*(SE)**	***P*-value**	***β_1_+ β_3_***	**Parameters of model fit**		
								**Dw**	**Root MSE**	***R* ^**2**^**
AURE (%)	−0.400 (0.193)	**0.044**	−4.817 (1.762)	**0.009**	0.308 (0.218)	0.164	−0.092	2.055	1.860	0.873
ACE/PCE (%)	−0.616 (0.102)	**0.000**	−2.492 (1.030)	**0.020**	0.465 (0.107)	**0.000**	−0.151	1.947	1.531	0.903
ACE per patient (yuan)	0.208 (0.385)	0.591	11.398 (3.841)	**0.005**	−0.630 (0.412)	0.133	−0.422	1.918	4.974	0.426

*AURE, antibiotic use rate of emergency patients; ACE, antibiotic cost of emergency patients; PCE, prescription cost of emergency patients; Dw, Durbin-Watson. Bold values mean P <0.05 and they are statistically significant*.

## Discussion

During and after the study period, the rate of nosocomial infection remained stable, and the rate of mortality mildly decreased, which indicates that the continued decline in antibiotic use is unlikely to negatively impact the quality of medical care provided.

The findings of this study demonstrate that antibiotic use of inpatients (AURI and AUD), outpatient (AURO), and emergency patients (AURE) notably decreased in the pre-intervention period from January 2011 to March 2012. Thus, it was apparent that soft measures such as systemic training and education play an important role in reducing antibiotic use. Similarly, a study by Onorato et al. (6) also confirmed the positive impact of a persuasive and educational AMS program on antibiotic use in ICUs in Italy. Although an intensified intervention led to an instant reduction in antibiotic use in April 2012 as expected, an accelerated downward trend was not observed from April 2012 to December 2014. Instead, the declining trend gradually tapered during the post-intervention period. To our knowledge, this may be primarily attributed to the capacity for reduction at different stages. In the beginning, a high rate of prescription at baseline provides sufficient room for reduction. When a lower level of antibiotic use is achieved, there is little room for further reduction. Similar findings were also observed in other AMS reports. For instance, two studies found no significant change in antibiotic use in the hospitals of women and children following AMS because of the low base levels of antibiotic use in these institutions (26, 27).

The impact of intensified CSRA policy on antibiotic expenditure (AE/ME, AE per patient, and ACE/PCE) is almost the same as that of antibiotic use. The growth trajectories of the cost indicators changed from a steep descending trend to a softened descending trend. As stated above, there is little room for a further decline in the later stages of intervention. We were even delighted to see that the downward trend of ACO/PCO sharpened after the intensified CSRA policy was implemented. But to our surprise, ACO per patient increased by 36.05 yuan and ACE per patient increased by 5.51 yuan in pre- post-comparisons. In the ITS analysis, ACO per patient also greatly increased prior to the full implementation of the intensified CSRA policy, which is inconsistent with other indicators. Maybe we can interpret this phenomenon from the policy itself. The CSRA policy only imposed quotas of AUR and AUD, but no claims were made about the cost of antibiotics. Thus, physicians might prescribe expensive antibiotics to ease the impact of the policy in the short term. Additionally, drug price inflation may also contribute to the increase of antibiotic cost during the whole study period. Similar findings have been reported in other studies where antibiotic expenditure fluctuated before reaching a stable status over a prolonged period (28, 29). Encouragingly, the upward trend of ACO per patient greatly softened from β = 1.965 to β = 0.320 after the intensified intervention, and the upward trend of ACE per patient reversed from β = 0.208 to β = −0.442 after the intensified intervention. They all demonstrated that intensified CSRA policy showed a positive effect on reducing antibiotic expenditure.

After 4 years of CSRA implementation in this hospital, AURO decreased from 25 to 22%, and AURI decreased from 65 to 54% in the last month of the study period. These two indicators are quite close to the goals set by the CSRA policy. However, AURE gradually approached 55% and AUD approached 52 DDDs/100PD in the last month of the study period, which far exceeded target values (AURE of 40% and AUD of 40DDDs/100PD). This begs several questions: are the goals of AURE and AUD set by the CSRA policy reasonable? To what extent is further reduction achievable without endangering the quality of care in this hospital? In China, data from 114 general tertiary-care hospitals in 30 provinces showed that the rate of antibiotic prescription was 27.82% in emergency departments in 2016 (30). One study showed that the level of AUD decreased to 49.5 DDDs/100 PD after CSRA in a general tertiary hospital, which is similar to our finding (31). Another study showed an excellent outcome of decreased antibiotic consumption (28.08 DDDs/100PD) following the implementation of CSRA policy, far below the target (2). These initial results were quite promising, and motivated us to continue working on reducing AURE and AUD.

## Limitations

We acknowledge that there are some limitations to our study. First, this study was a single-center study without a control group. Although ITS analysis was applied to minimize the threats to internal validity, we still could not ensure that CSRA policy implementation was the only reason for the changes we report in our findings. A concurrent control hospital can be implemented in future studies to account for simultaneous secular trends in antibiotic use (32). Second, the indicators in this study do not fully encompass the possible impacts of CSRA policy. Reducing antibiotic use and expenditure may reflect reductions in overuse, but we cannot be certain that these observed changes reflect better quality antibiotic prescription. Third, we did not examine disease-specific antibiotic use or expenditure. However, different disease-specific factors might have different effects on these outcomes. For different specific diseases, there are differences in treatment modality, infection prevention, and antibiotic usage. These findings should be interpreted with caution due to these limitations.

## Conclusion

In summary, this study provides detailed ITS analysis of the impact of CSRA policy (soft measures and intensified rigid measures) on antibiotic use in a single teaching hospital. Implementation of CSRA policy was associated with declining antibiotic use and antibiotic expenditure in inpatients, outpatients, and emergency patients. However, it is also important to note that the declining trend of antibiotic consumption slowed down due to the limited capacity for a decline in the later stages of CSRA intervention.

## Data Availability Statement

The original contributions presented in the study are included in the article/supplementary material, further inquiries can be directed to the corresponding author/s.

## Ethics Statement

Institutional Ethics Committee approval was obtained from the Nanjing Medical University Ethics Committee (Grant number: ethical review 201236). Due to this being a retrospective study, the patient data were aggregated and the individual patients' information was not reported in this study. Therefore, the study was approved with a waiver for informed consent.

## Author Contributions

XQ and XL initiated the study concept. XQ conducted data analysis and wrote the first draft of the manuscript. YP and JG collected the data of inpatients. SX and YL led the data collection of outpatients and emergency patients. XL and DS contributed to the data analysis and interpretation of the data and also revised the draft of the manuscript. All the authors read and approved the final manuscript.

## Funding

This study was supported by the National Natural Science Foundation of China (Grant No: 71673147), the Jiangsu Pharmaceutical Association Aosaikang Hospital Pharmaceutical Fund (Grant No: A201621), the China Medical Board (Grant No: 17-277), and the Joint Project between Southeast University and Nanjing Medical University (Grant No: 2018DN0023).

## Conflict of Interest

The authors declare that the research was conducted in the absence of any commercial or financial relationships that could be construed as a potential conflict of interest.

## Publisher's Note

All claims expressed in this article are solely those of the authors and do not necessarily represent those of their affiliated organizations, or those of the publisher, the editors and the reviewers. Any product that may be evaluated in this article, or claim that may be made by its manufacturer, is not guaranteed or endorsed by the publisher.
